# Determination of ertapenem in plasma and ascitic fluid by UHPLC-MS/MS in cirrhotic patients with spontaneous bacterial peritonitis

**DOI:** 10.1515/almed-2023-0168

**Published:** 2023-12-20

**Authors:** Raúl Rigo-Bonnin, Alberto Amador, María Núñez-Gárate, Virgínia Mas-Bosch, Ariadna Padullés, Sara Cobo-Sacristán, José Castellote

**Affiliations:** Clinical Laboratory, Bellvitge University Hospital, Bellvitge Biomedical Research Institute (IDIBELL), Barcelona, Spain; Service of Pharmacy, Bellvitge University Hospital, Bellvitge Biomedical Research Institute (IDIBELL), Barcelona, Spain; Unit of Hepatology and Liver Transplant, Service of Gastroenterology, Bellvitge Biomedical Research Institute (IDIBELL), Barcelona, Spain

**Keywords:** ertapenem, ascitic fluid, plasma, UHPLC-MS/MS

## Abstract

**Objectives:**

Spontaneous bacterial peritonitis is a frequent severe complication in cirrhotic patients with ascites. Carbapenem antibiotics are currently the treatment of choice for patients with hospital-acquired or healthcare-related infections. However, there is limited evidence available on the efficacy of ertapenem in cirrhotic patients with spontaneous bacterial peritonitis. As a result, the pharmacokynetics and pharmacodynamics of this antibiotic are still unknown. The objective of this study was to develop and validate measurement procedures based on liquid chromatography-tandem mass spectrometry (UHPLC-MS/MS) to determine ertapenem concentrations in plasma and ascitic fluid.

**Methods:**

Samples were pretreated by acetronile protein-precipitation. Chromatographic separation is performed on a C_18_ reversed-phase Acquity^®^-UPLC^®^-BEH^TM^ column (2.1 × 100 mm id, 1.7 µm) using a non-linear gradient of water/acetonitrile containing 0.1 % of formic acid at a flow rate of 0.4 mL/min. Ertapenem and its internal standard (ertapenem-D_4_) are detected by tandem mass spectrometry using positive electrospray ionization and multiple reaction monitoring, and using 476.2 → 346.0/432.2 as mass transition for ertapenem and 480.2 → 350.0 for its internal standard.

**Results:**

No significant interferences or carry-over contamination were observed. Imprecisions, absolute relative bias, matrix effects and normalized recoveries were ≤14.5 %, ≤9.3 % (92.8–104.5) % and (98.8–105.8) %, respectively. Chromatographic measurement procedures were linear from (0.50–100) mg/L.

**Conclusions:**

The measurement procedures based on UHPLC-MS/MS developed and validated in this study could be useful in pharmacokynetic and pharmacodynamic studies in subjects with liver cirrhosis who develop spontaneous bacterial peritonitis treated with ertapenem.

## Introduction

Spontaneous bacterial peritonitis (SBP) is a frequent complication in patients with cirrhosis. This event is defined as ascitic fluid infection in cirrhotic patients, in the absence of a secondary cause. Diagnosis is established by parecentesis demonstrating >250 polymorphonuclear cells in ascitic fluid with or without a positive culture [1, 2]. Gram-negative bacteria are most frequently found. However, in the recent years, there are a growing number of positive cultures for multiresistant bacteria, which complicates treatment and increases morbimortality in cirrhotic patients [3–6]. Current guidelines recommend treating hospital-acquired or healthcare-related SBP with carbapenems, either in monotherapy or in combination with glycopeptide, respectively [1, 2, 7].

Ertapenem (ETP) is a carbapenemic antibiotic indicated for the treatment of severe intra-abdominal infections and for infections caused by multiresistant enterobacteria [5, 7]. At pharmacokynetic level, the bactericidal activity of ETP is time-dependent, and the pharmacokynetic/pharmacodynamic index that best correlates with its efficacy is the percentage of time that the unbound antibiotic concentration (‘free’) exceeds the minimal inhibitory concentration or MIC (*f*T>MIC) between two consecutive doses. This percentage is assumed to be close to 50 %. However, in particular contexts (i.e. in resistant microorganism infections with elevated MICs), longer time intervals may be required (70–100) [8, 9].

Several studies have measured plasma ETP concentrations by high-pressure liquid chromatography ((U)HPLC) coupled to tandem mass spectrometry (MS/MS) [10], [11], [12], [13], [14], [15], [16], [17]. To the best of our knowledge, this is the first study to measure ETP concentrations in ascitic fluid in cirrhotic patients with SBP. The objective of this study was to develop and validate measurement procedures based on UHPLC-MS/MS to determine ETP concentrations in plasma and ascitic fluid.

## Materials and methods

### Reagents

Certified reference materials included Alsachim Ertapenem sodium salt (Cat. no. C3377;98.2 % purity) and [^2^H_4_]-Ertapenem sodium salt (Cat.no. C3376; 95.5 % purity; 98.5 % isotopic enrichment) (Illkirch Graffenstaden, France).

Solvents included acetonitrile, formic acid, water, and LC-MS/MS high-quality methanol from Merck Millipore Group (Darmstadt, Germany).

### Collection of plasma and ascitic fluid samples not containing ertapenem

We used plasma and ascitic fluid samples received at the Emergency Laboratory of our hospital. Blood and ascitic fluid samples were collected in Vacuette^®^ plasma 4-mL tubes containing lithium heparin (Greiner Bio-One GmbH, Kremsmünster, Austria). Tubes were centrifuged at 2000 *g* for 10 min at room temperature. The resulting supernatant was stored in polypropylene 2-mL tubes at (−75 ± 3) °C for later analysis. Previously, an aliquot of each biological fluid was obtained to confirm the absence of ETP using measurement procedures based on UHPLC-MS/MS.

### Preparation of calibration materials, internal quality control materials, and internal standard working solutions

Two primary ETP-containing solutions of 1 g/L are prepared from *Ertapenem sodium salt* material. The first solution is used to prepare calibration materials, whereas the second is used for internal quality control materials (QC). Then, several secondary aqueous solutions are prepared from the primary solution at values ranging (5–1,000) mg/L and stored protected from light at (−75 ± 3) °C. Every day, when patient samples are processed, nine ETP calibration materials are prepared at values of 0.50; 3.50; 7.50; 15.0; 25.0; 40.0; 60.0; 80.0 and 100 mg/L by diluting the corresponding secondary solutions with samples of plasma or ascitic fluid not containing ETP to a ratio of 1:9.

QCs are prepared and stored in similar conditions to those of calibration materials by using the other primary ETP solution. QCs of plasma and ascitic fluid are performed at values of 1.50; 10.0; 50.0 and 75.0 mg/L.

The primary internal standard (Ertapenem-D_4_) is prepared at 1 mg/L in methanol from *[* ^*2*^ *H*_*4*_ *]-Ertapenem sodium salt material*. This solution is stored protected from light at (−75 ± 3) °C. The internal standard (IS) working solution is prepared by adding 300 µL of the primary solution to 10 mL of acetonitrile (30 mg/L).

### Instrumentation

Tests were performed on an Acquity^®^-UPLC^®^ chromatograph coupled to a triple quadrupole mass spectrometer Acquity^®^-TQD^®^ (Waters, Milford, MA, EEUU).

### Chromatographic conditions

Chromatographic separation is performed using a reversed-phase Acquity Waters^®^-UPLC^®^ BEH™ C_18_ (2.1 × 100 mm id, 1.7 µm) column by maintaining the column compartment at 40 °C.

A gradient elution of the mobile phase composed of a solvent A (mobile phase A), composed of 0.1 % (v/v) formic acid in water, and a solvent B (mobile phase B) containing 0 0.1 % (v/v) formic acid in acetonitrile (Table 1). Temperature of the autosampler is kept at (15 ± 1) °C.

**Table 1:** Chromatographic elution.

Step	Total time, min	Mobile phase A, %	Mobile phase B, %	Rate, mL/min	Type of elution
1	0.0	98	2	0.4	Isocratic
2	0.4	50	50	0.4	Linear gradient
3	2.0	98	2	0.4	Non-lineal gradient

Mobile phase A: Formic acid 0.1 % (v/v) in water. Mobile phase B: Formic acid 0.1 % (v/v) in acetonitrile.

### Mass spectrometry conditions

Generic parameters of the mass spectometer for ETP and its IS included: capillary potential, 1.5 kV; extractor source potential, 3 V; radio frequency lense potential, 0.1 V; ion source temperature, 125 °C; desolvation temperature, 450 °C; desolvation gas flow, 800 L/h; cone glas flow, 50 L/h; and collision gas flow, 0.20 mL/min. Nytrogen is used as the nebulization and desolvation gas, and argon as collision gas.

Detection of ETP and its IS (ETP-D_4_) is performed by multiple reaction monitoring (MRM) and positive electrospray ionization (ESI+) to verify the formation of the adduct [ETP-H]^+^. For ETP, we use a mass transition of 476.2 → 346.0 for quantification, and 476.2 → 432.2 for qualification. For ETP-D_4_, only the transition 480.2 → 350.0 is used. Cone potential is 25 V for ETP and its IS. Collision energies are 15 eV for the first transition of ETP and its IS, and 25 eV for the second transition. The dwell time for each compound is selected in 100 ms.

### Pretreatment of samples

A total of 100 µL of calibration materials, QC, plasma/ascitic fluid samples were transferred to microcentrifuge polypropylene 1.5 mL tubes. Next, protein precipitation is induced by adding 300 μL of the working solution of the IS, following by 2 min homogenization in a vortex, and 5 min centrifugation at 11,000 *g* at room temperature. Then, 100 μL of the resulting supernatant are diluted with 400 μL of the mobile phase A, the mixture is shaken in a vortex for 5 s and 10 μL are injected in the chromatograph.

### Validation study

The measurement procedures developed based on the UHPLC-MS/MS were validated following the guidelines of the European Medicines Agency (EMA) [18] and the Clinical and Laboratory Standards Institute (CLSI) [19].

### Selectivity

The selectivity study involves the use of double blank samples (not containing ETP or its IS); blank samples (only containing IS); samples close to the lower limit of quantification (LLOQ) and 18 different samples of plasma and ascitic fluid of patients not treated with ETP received in the emergency laboratory. Turbidity was visible in six plasma samples (lipemia indices with values 114 to 395); six were hemolyzed (hemolysis indices ranging 27 to 165); whereas no apparent interference was observed in another six samples (hemolysis and lipemia indices below 6 and 12, respectively). Hemolysis was visible in four samples of ascitic fluid (hemolysis indices within 20 and 150); turbidity was visible in two (lipemia indices of 103 and 202) and interference was visible in 10 samples (indices of hemolysis and lipemia below 8 and 15, respectively).

Hemolysis and lipemia indices were obtained by processing specimens on a Cobas^®^ 6,000 de Roche Diagnostics (Risch-Rotkreuz, Suiza) analyzer.

The absence of interfering components was confirmed if the area under the curve (AUC) of the peak of all potential interfering peaks for each sample processed during the ETP retention time was <20 % of the AUC for the samples close to the LIC for ETP, and 5 % for the IS (ETP-D_4_) [18].

### Specificity

The specificity and the sensitivity study involved the use of similar methods but different patient samples. The specificity study was performed using 10 different samples of plasma and ascitic fluid of polypharmacy patients receiving antibiotic therapy with ampicillin, aztreonam, cloxacillin, cefepime, ceftazidime, ceftriaxone, daptomycin, dalbavancin, meropenem and piperacillin.

The absence of interfering components was confirmed if the area under the curve (AUC) of the peak of all potential interfering peaks for each of the samples processed during the ETP retention time was <20 % of the AUC for the samples close to the LIC for ETP, and 5 % for the IS (ETP-D4) [18].

### Matrix effect and recovery of pretreated samples

To evaluate the matrix effect (ME) and recovery effect (RE) in plasma and ascitic fluid, three pools of different samples were used at values of 1.50, 10.0, 50.0 and 75 mg/L for ETP, and at a value of 30 mg/L for the IS. These three pools of samples included primary ETP solutions (and their IS) diluted with the mobile phase A (Samples A); six samples of plasma (and ascitic fluid) of different patients not containing ETP to which this antibiotic was added after the protein precipitation process (Samples B); and the same six samples to which ETP is added prior to protein precipitation (Samples C). These pools of samples are randomly processed five times.

The ME and RE is calculated (in %) as:

$$\text{ME }\left(\%\right)=\frac{{\text{Mean}\;\text{AUC}}_{\text{Samples}\;B}}{{\text{Mean}\;\text{AUC}}_{\text{Samples}\;A}}\cdot 100$$

$$\text{RE }\left(\%\right)=\frac{{\text{Mean}\;\text{AUC}}_{\text{Samples}\;C}}{{\text{Mean}\;\text{AUC}}_{\text{Samples}\;B}}\cdot 100$$

The normalized ME and RE are also calculated by dividing the ME and RE values obtained for ETP by the ME and RE values obtained for the IS.

The relative bias should fall within the ±15 % interval with respect to the nominal concentration, and imprecision cannot exceed 15 % [18]. Variation of the ME and RE obtained for all concentrations evaluated should be <15 % [19].

### Calibration curve

The integrated AUCs of smooth peaks, calibration curves, and estimated ETP concentrations were performed using MassLynx™ v4.1 (Waters) software.

Calibration curves were generated by linear adjustment of the AUC-ETP/AUC-ETP-D coefficient_4_ multiplied by the ETP-D_4_concentration (y-axis), against the nominal ETP concentration (x-axis). Linear regressions are estimated by 1/X weighting by excluding the option of forcing the calibration curve through the origin.

All estimated concentrations of calibration materials should fall within the ±15 % interval with respect to their corresponding nominal concentrations (±20 % in the case of a concentration close to the LLOQ) [18].

### Precision and trueness

All QCs prepared are used to estimate intra- and inter-day precision (expressed as a coefficient of variation, CV) and trueness (expressed as relative bias, *δ*_*r*_). 30 aliquots of each QC are repeatedly processed in the same run the same day, and for 60 non-consecutive days over a month and a half, respectively.

To calculate CV and *δ*_*r*_ at values close to the LLOQ, the same procedure is performed, but with materials prepared at concentrations close to 0.50 mg/L.

The CV obtained should be ≤15 %, whereas *δ*_*r*_ should be ±15 %. In the case of the LLOQ, the signal/noise (S/N) ratio should be ≥5 and have a CV≤20 % and a *δ*_*r*_ *δ*_*r*_ of ±20 % [18].

### Carry-over contamination

Carry-over contamination was evaluated by processing double blanks (of plasma and ascitic fluid), samples with values close to the LLOQ, and the calibration materials with the highest concentrations, by the following order:

Sample with ETP values close to the LLOQ − calibration material with the highest concentration− double blank

Carry-over contamination was acceptable if the AUC of the peak ETP in double blanks did not exceed 20 % of the AUC of the peak ETP of the sample in the LLOQ, and 5 % of the AUC of the peak of the IS [18].

### Integrity of the dilution

To assess the integrity of the dilution, the samples of plasma and ascitic fluid are prepared at values exceeding two times the ULOQ; then, samples are processed six times and diluted 10 times with the corresponding blank. Next, the mean value obtained is compared against the nominal concentration.

The CV should be ≤15 % and *δ*_*r*_±15 % [18].

### Stability

To evaluate the stability of primary and secondary solutions, they are diluted with the mobile phase A; then, the resulting solutions are stored at (−75 ± 3) °C for analysis three months later.

The stability of pretreated samples on the autosampler is assessed by reinjecting them at 6 and 24 h after they are stored at (15 ± 1) °C.

Short- and long-term stability studies are carried out using ETP QCs. To evaluate short-term stability, QCs are stored at room temperature for 2, 4 and 6 h, and subsequently processed. To assess long-term stability, samples are frozen at (−75 ± 3) °C and processed at one month.

In all cases, and using 10 replicates, stability is estimated as a function of the deviation percentage (D%) of the mean value obtained with respect to the nominal concentration:

$$D\%=\frac{\text{Mean}\;\text{value}\;\text{obtained}\;\text{in}\;\text{normal}\;\text{replicated}-\text{Nominal}\;\text{concentration}}{\text{Nominal}\;\text{concentration}}\cdot 100$$

The D% obtained should fall within the ±15 % interval [18].

### Clinical application

The measurement procedures based on UHPLC-MS/MS/MS described above were developed and validated for use in a research study on the efficacy of ETP therapy in cirrhotic patients with SBP. This study was approved by the Institutional Review Board (JCA-ERT-2016-01) and in compliance with the tenets of the World Medical Association and the Declaration of Helsinki.

### Patients

Our cohort was composed of patients with a diagnosis of hepatic cirrhosis with hospital-acquired or healthcare-related SBP admitted to the Unit of Gastroenterology of our hospital.

### Ertapenem administration

Patients received an intravenous infusion of ETP at a dose of 1 g/24 h for 30 min. The duration of treatment ranges from 5 to 7 days, based on culture results, and prior cytological and microbiological confirmation that infection had disappeared.

### Sample collection

As ertapenem reaches a steady-state at 20 h from the initiation of treatment [20], patient samples are collected at 48 h, 60 h, 72 h, 84 h, 96 h, 108 and 120 h after the initiation of treatment. Conversely, samples of ascitic fluid are collected at 48 h, 72 h, 96 h and 120 h after the initiation of treatment. This way, the different concentrations of “free” ertapenem at steady-state (*f*ETPss) are obtained. To calculate *f*ETPss, a 95 % protein binding is assumed for ETP [21].

Plasma samples were obtained by venipuncture, whereas samples of ascitic fluid were obtained by paracentesis, according to standard methods. Samples were collected and processed as described above.

## Results and discussion

### Development of measurement procedures based on UHPLC-MS/MS

**Figure 1:**
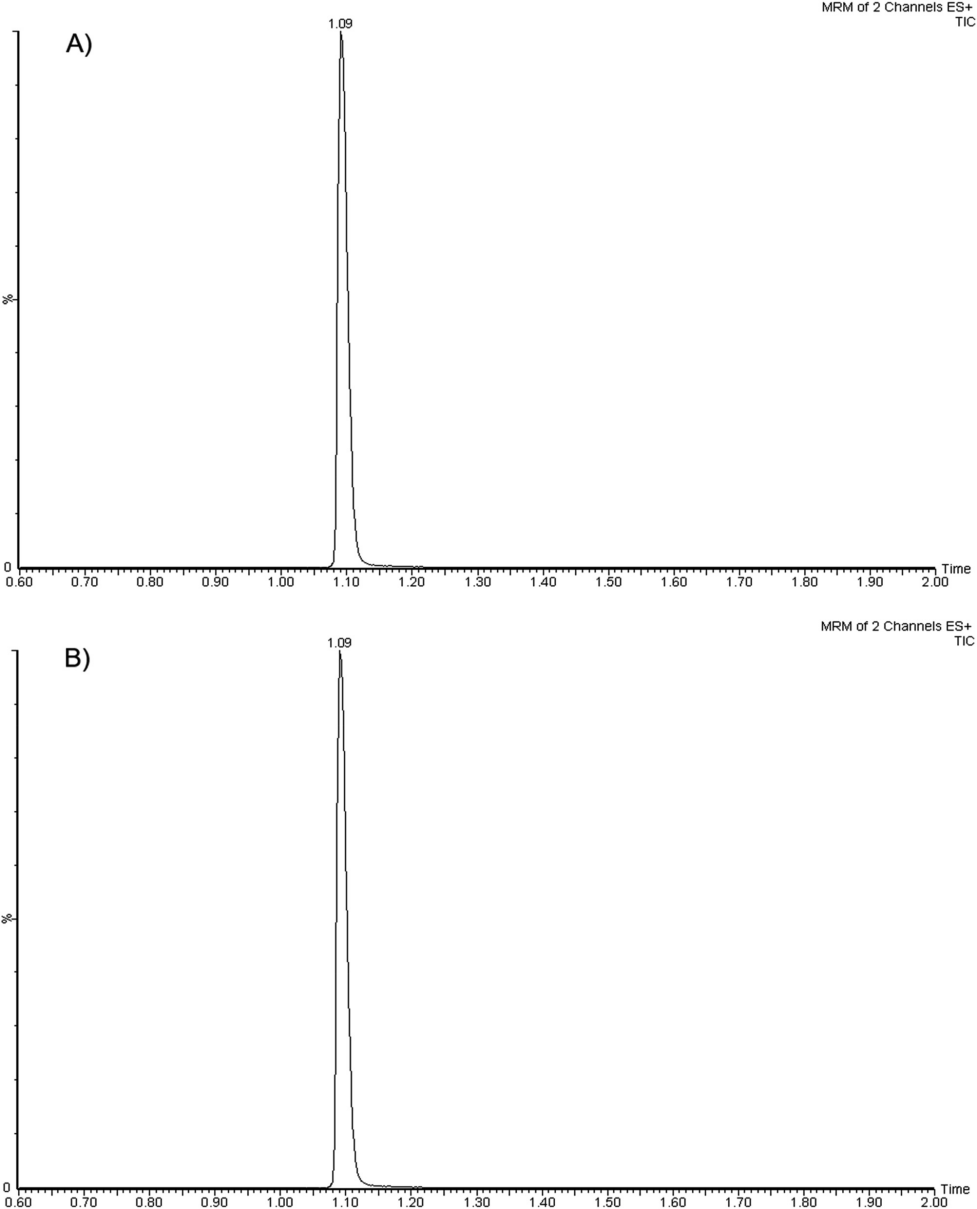
Chromatograms showing ertapenem concentrations (A) in plasma (B) and ascitic fluid in a cirrhotic patient with spontaneous bacterial peritonitis receiving intravenous ertapenem at a dose of 1 g/12 h for 30 min.

Retention times for ETP and its IS are 1.09 min, with a total chromatographic time of 3.0 min, being the two values constant and reproducible (Figure 1). In most of the procedures published, the total chromatographic time ranges from 2 to 15 min, which are similar or higher than in our procedure [15], [16], [17], [18], [19], [20], [21], [22].

### Validation of measurement procedures based on UHPLC-MS/MS

#### Selectivity and specificity

None of the samples processed showed interfering peaks with significant AUCs in retention times of ETP (values ranged from 0.2 to 1.0 %) and its IS (values ranged from 0.0 to 0.3 %).

#### Matrix and recovery effect

Table 2 shows the results obtained for the ME and RE. The CV and *δ*_*r*_ obtained ranged from (4.3–11.0) % and (2.3–8.0) %, respectively.

**Table 2:** Matrix effects, normalized matrix effects, recoveries and normalized recoveries for ertapenem concentration in plasma and ascitic fluid.

Analyte	Nominal value, μg/L	Value obtained, μg/L	Matrix effect (%)	Normalized matrix effect (%)	Recovery (%)	Normalized recovery (%)
Pla – Ertapenem; mass c.	1.50	1.54	87.8 (11.9)	104.5 (12.6)	71.5 (11.0)	99.0 (12.4)
	10.0	10.5	85.3 (9.5)	101.5 (10.9)	73.2 (9.0)	101.4 (9.5)
	50.0	51.5	83.8 (7.9)	99.8 (8.4)	75.9 (8.0)	105.1 (8.8)
	75.0	76.7	81.2 (6.3)	96.7 (7.5)	76.4 (6.5)	105.8 (7.2)
AF – Ertapenem; mass c.	1.50	1.56	92.5 (9.9)	100.8 (10.3)	70.3 (9.5)	98.8 (10.1)
	10.0	10.8	90.3 (7.6)	98.4 (8.4)	71.7 (8.2)	100.7 (8.8)
	50.0	53.6	87.4 (5.9)	95.2 (6.5)	72.6 (5.4)	102.0 (6.2)
	75.0	78.2	85.2 (4.5)	92.8 (5.8)	74.5 (4.3)	104.6 (5.0)

Coefficients of variation (shown between brackets in %) between the different samples selected, which were processed five times each. Following IUPAC and IFCC recommendations [22]: AF, ascitic fluid; Pla, plasma; mass c., mass concentration.

The IS chosen (ETP-D_4_) makes it possible to make up for the loss of ETP during sample pretreatment, independently from the value of the analyte, and with an acceptable imprecision (≤15 %). With regard to ME values, ionic suppression was observed in ETP, which is compensated with the use of the IS.

#### Calibration curve

All calibration curves obtained showed a linear regression model within the established measurement interval (0.50–100) mg/L. Coefficients of determination exceeded 0.9945.

The deviation percentages obtained for the estimated ETP concentrations in calibration materials with respect to their nominal concentrations ranged from 5.1 to 13.1 % for plasma calibrators, and from 5.3 to 7.9 % for ascitic fluid calibrators.

#### Precision and trueness

CV and *δ*_*r*_ fell within the (3.9–17.3) % and (3.9–16.0) %, respectively (Table 3). Moreover, the *S/N* ratios obtained for LLOQ were ≥10.5 for plasma ETP concentrations, and ≥12.2 for ETP concentration in ascitic fluid.

**Table 3:** Values of imprecision and relative intra- and interday bias for the measurement procedures based on UHPLC-MS/MS that enable determination of ertapenem in plasma and ascitic fluid.

Analyte Nominal concentration, µg/L (type of sample)	Intra-day (n=30)				Inter-day (n=30)		
	$\bar{X}$, µg/L	CV %	*δ*_r_, %		$\bar{X}$ ± *s*, µg/L	CV, %	*δ*_r_, %
Pla – Ertapenem; mass c.							
0.50 (LLOQ)	0.55	14.2	10.0		0.58	17.3	16.0
1.50 (QC1)	1.61	10.9	7.3		1.64	14.5	9.3
10.0 (QC2)	10.5	7.2	5.0		10.9	10.7	9.0
50.0 (QC3)	52.5	5.5	5.0		54.2	7.8	8.4
75.0 (QC4)	77.2	4.7	2.9		80.2	5.9	6.9
AF – Ertapenem; mass c.							
0.50 (LLOQ)	0.52	12.1	4.0		0.53	15.4	6.0
1.50 (QC1)	1.58	8.7	5.3		1.55	11.6	3.3
10.0 (QC2)	10.2	6.5	2.0		10.5	8.9	5.0
50.0 (QC3)	51.4	4.8	2.8		52.0	7.1	4.0
75.0 (QC4)	76.3	3.9	1.7		77.9	5.5	3.9

n, number of materials processed; $\bar{X}$, mean; CV, coefficient of variation; *δ*_r_, relative bias; LLOQ, lower limit of quantification; QC, interrnal quality control. Following IUPAC and IFCC recommendations [22]: AF, ascitic fluid; Pla, plasma; mass c., mass concentration.

The LLOQ, CV and *δ*_*r*_ obtained for plasma ETP concentrations are similar or lower than the ones obtained using other measurement methods described elsewhere [15], [16], [17], [18], [19], [20], [21], [22].

#### Carry-over contamination

There were no chromatographic peaks with significant AUCs in the same ETP and IS retention time. For ETP, the coefficients¡ of ACUs of chromatographic peaks obtained with respect to AUCs at values close to the LLOQ was 2.2 % for plasma and 4.1 % for ascitic fluid. Additionally, for the IS, coefficient of AUCs were 0.6 and 1.0 %.

#### Integrity of the dilution

The CV obtained in the study of dilution integrity (dilution 1/10) were 3.8 % for plasma samples, and 2.8 % for samples of ascitic fluid. Moreover, *δ*_*r*_ values were −1.8 % and −2.2 %, respectively.

#### Stability

Primary and secondary solutions are stable for at least three months at (−75 ± 3) °C (D% ≤−3.9 % and ≤−4.3 %, respectively). With regard to the other stability studies performed, ETP concentrations are stable for 6 h at room temperature (D% ≤−10.7 % for plasma and ≤−12.4 % for ascitic fluid), and for 3 months when stored at (−75 ± 3) °C (D% ≤−6.9 % for plasma and ≤−8.8 % for ascitic fluid). ETP concentrations also remained stable on the autosampler for 12 h at (15 ± 1) °C (%D ≤−13.8 % for plasma and ≤−14.5 % for ascitic fluid).

#### Clinical application

ERT concentrations in plasma and ascitic fluid in cirrhotic patients with SBP are shown in Table 4.

**Table 4:** Characteristics of cirrhotic patients with ascites with spontaneous bacterial peritonitis and ertapenem concentrations in plasma and ascitic fluid at different time points.

Subject	Sex	Age, years	Weight, kg	GF, mL/min	Isolated organism	MIC^a^, mg/L	Analyte (results in mg/L)	48 h	60 h	72 h	84 h	96 h	108 h	120 h
1	Male	55	55	>90	*Staphylococcus aureus*	0.016	Pla – ETPss; mass c mass c	36.6	40.2	31.2	28.8	32.8	26.7	30.2
							Pla *– f*ETPss; mass c	1.81	2.01	1.56	1.44	1.64	1.34	1.51
							AF *–* ETPss; mass c	18.7	–	23.0	–	23.4	–	26.6
							AF *– f*ETPss; mass c	0.935	–	1.15	–	1.17	–	1.33
2	Male	62	82	>90	*Staphylococcus aureus*	0.030	Pla – ETPss; mass c	5.44	6.66	8.08	5.83	5.01	–	–
							Pla – fETPss; mass c	0.272	0.333	0.404	0.292	0.251	–	–
							AF – ETPss; mass c	0.92	–	1.33	–	0.99	–	–
							AF – fETPss; mass c	0.046	–	0.067	–	0.050	–	–
3	Male	55	101	70	*Enterobacter cloacae*	0.125	Pla – ETPss; mass c	33.4	34.2	33.2	34.5	37.3	35.4	–
							Pla – fETPss; mass c	1.65	1.71	1.66	1.73	1.87	1.77	–
							AF – ETPss; mass c	3.21	–	3.54	–	4.01	–	–
							AF – fETPss; mass c	0.161	–	0.177	–	0.201	–	–
4	Female	65	65	82	*Klebsiella pneumoniae*	0.060	Pla – ETPss; mass c	4.52	5.24	7.28	8.05	12.2	18.1	–
							Pla – fETPss; mass c	0.226	0.262	0.364	0.403	0.610	0.905	–
							AF – ETPss; mass c	1.79	–	2.22	–	4.48	–	–
							AF – fETPss; mass c	0.090	–	0.111	–	0.224	–	–
5	Female	54	54	>90	*Escherichia coli*	0.250	Pla – ETPss; mass c Pla – fETPss; mass c	9.11 0.455	8.22 0.411	8.38 0.419	7.34 0.367	7.54 0.377	8.83 0.442	9.21 0.461
							AF – ETPss; mass c	7.14	–	5.94	–	5.64	–	5.93
							AF – fETPss; mass c	0.357	–	0.297	–	0.282	–	0.297

GF, estimated glomerular filtration rate according to the CKD-EPI formula; MIC, minimal inhibitory concentration; ETPss, ertapenem in steady state; *f*ETPss, unbound (“free”) ertapenem at steady state. ^a^MIC values for ertapenem are determined by the E-test^®^ method (bioMérieux, Marcy-l’Étoile, France). According to IUPAC and IFCC recommendations [22]: AF, ascitic fluid; Pla, plasma; mass c, mass concentration.

The results obtained reveal that 100 % of the *f*ETPss reach the target interval (50–100) % *f*T>CMI. The efficacy of the antibiotic treatment, defined as cytological and microbiological confirmation of the absence of infection was achieved in 5–6 days for all patients.

## Conclusions

This study was conducted to develop and validate measurement procedures based on UHPLC-MS/MS for the determination of ETP concentrations in plasma and ascitic fluid. The results obtained confirm the acceptable selectivity, specificity, power of detection, accuracy, trueness and precision of the procedures developed. Therefore, they can be used in PK/PD research studies. In addition, these methods can be used for ETP pharmacotherapeutic monitoring in routine clinical practice.
